# Biosynthesis of Secondary Metabolites Based on the Regulation of MicroRNAs

**DOI:** 10.1155/2022/9349897

**Published:** 2022-03-04

**Authors:** Rajib Hossain, Cristina Quispe, Abu Saim Mohammad Saikat, Divya Jain, Arslan Habib, Pracheta Janmeda, Muhammad Torequl Islam, Radha, Sevgi Durna Daştan, Manoj Kumar, Monica Butnariu, William C. Cho, Javad Sharifi-Rad, Aliya Kipchakbayeva, Daniela Calina

**Affiliations:** ^1^Department of Pharmacy, Life Science Faculty, Bangabandhu Sheikh Mujibur Rahman Science and Technology University, Gopalganj 8100, Bangladesh; ^2^Facultad de Ciencias de la Salud, Universidad Arturo Prat, Avda. Arturo Prat 2120, Iquique 1110939, Chile; ^3^Department of Biochemistry and Molecular Biology, Life Science Faculty, Bangabandhu Sheikh Mujibur Rahman Science and Technology University, Gopalganj 8100, Bangladesh; ^4^Department of Bioscience and Biotechnology, Banasthali Vidyapith, Rajasthan, India; ^5^Lab of Infectious and Molecular Immunology, School of Life Sciences, Fudan University, Shanghai, China; ^6^School of Biological and Environmental Sciences, Shoolini University of Biotechnology and Management Sciences, Solan 173229, India; ^7^Department of Biology, Faculty of Science, Sivas Cumhuriyet University, Sivas 58140, Turkey; ^8^Beekeeping Development Application and Research Center, Sivas Cumhuriyet University, Sivas 58140, Turkey; ^9^Chemical and Biochemical Processing Division, ICAR-Central Institute for Research on Cotton Technology, Mumbai 400019, India; ^10^Banat's University of Agricultural Sciences and Veterinary Medicine “King Michael I of Romania” from Timisoara, Timisoara, Romania; ^11^Department of Clinical Oncology, Queen Elizabeth Hospital, Kowloon, Hong Kong; ^12^Facultad de Medicina, Universidad del Azuay, Cuenca, Ecuador; ^13^Faculty of Chemistry and Chemical Technology, Al-Farabi Kazakh National University, Almaty 050040, Kazakhstan; ^14^Department of Clinical Pharmacy, University of Medicine and Pharmacy of Craiova, Craiova 200349, Romania

## Abstract

MicroRNA (miRNA), a noncoding ribonucleic acid, is considered to be important for the progression of gene expression in plants and animals by rupture or translational repression of targeted mRNAs. Many types of miRNA regulate plant metabolism, growth, and response to biotic and abiotic factors. miRNA characterization helps to expose its function in regulating the process of post-transcriptional genetic regulation. There are a lot of factors associated with miRNA function, but the function of miRNA in the organic synthesis of by-products by natural products is not yet fully elucidated. The current review is aimed at observing and characterizing miRNAs and identifying those involved in the functioning of the biosynthesis of secondary metabolites in plants, with their use in controlled manipulation.

## 1. Introduction

miRNAs are eighteen to twenty-eight nucleotides containing single-stranded RNA molecules which are not transcribed into proteins during transcription. It takes part in post-transcriptional regulation by binding to the messenger RNA and inhibiting the expression of specific genes [1]. These types of molecules are usually expressed in eukaryotes such as animals and plants and some viruses [2, 3].

The first miRNA was reported in *Caenorhabditis elegans* is *Lin-4*; then, further studies identify more than 18,226 other types of miRNAs in the same organisms such as 22 (nt) lin-4 and 21 (nt) let-7 [4]. While miRNA-targeted gene interconnections are extensively prolonged, this technique is restrained among domains [5]. miRNAs are disbursed in genetic material as clumps exhibit as a polycistronic segment with features [6]. Most miRNAs in flora are encrypted with their prime transcript and some precedents of miRNA clumps described as miR395. The basic identified hotspots for their beginning are introns [7]. Most of the processes in which they affect are the expansion of time and host-pathogen connection in addition to cell differentiation, proliferation, apoptosis, and tumorigenesis.

In eukaryotes and metazoans, miRNAs are a form of noncoding (22-nucleotide) ribo regulators that control gene expression which has an exciting role in the biosynthesis of plant secondary metabolites like flavonoids, terpenoids, alkaloids, and some other compounds [8, 9]. RNA polymerase II is a precursor RNA also described as the pri-miRNA that helps to stimulate the production of miRNA, processed by DICER-LIKE 1 (DCL1) to fully grown or developed miRNA [8].

In this review, we have updated the knowledge about the present understanding of miRNA-based regulation of biosynthesis and accumulation of secondary metabolites in plants. The data written in English were collected, using scientific search engines such as PubMed/Medline, ScienceDirect, Web of Science, Scopus, and Google Scholar. The search terms used were microRNA, biosynthesis, secondary metabolites, miRNA, flavonoids, alkaloids, and terpenoids.

The scientific names of the plants have been validated according to the Plant List [10, 11].

## 2. miRNA as a Secondary Metabolite Regulator

The polyadenylated caps and RNAs are the precursor molecules for the synthesis of miRNA both in plants and animals, and RNA polymerase II (RNAPII) transcribed many coding RNAs, although in plants, nucleus RNAase dicer-like 1 (DCL1) and their essential proteins SERRATE (SE) and hyponastic leaves (HYL1) synthesize primary miRNA but the Drosha gene is not found in plants [5, 12]. In *Arabidopsis thaliana*, the precursor miRNA, dsRNA, is synthesized in the nucleus by the splitting of pri-miRNA by HYL1 and DCL1. The second splitting is done by the activity of HYL1 and DCL1 which results in releasing of miRNA; methylation of two nucleotides 3′ overhangs is performed by methyltransferase HUA enhancer1 (HEN1). Adult single-stranded cytoplasmic miRNA activated by AGO1 is found in the RISC (RNA-induced silencing complex) ribonucleoprotein and by breaking mRNA suppresses its function [13]. The miRNA binds with mRNA at the 3′ untranslated region (3′ UTR) which inhibits the activity of miRNA [14]. The transcriptional factors regulate the expression of miRNA. The transcription of miRNA genes and effective use of DCL1 in the biosynthesis of miRNA is facilitated by At-Negative on TATA-less 2 (NOT2) [15]. Cell division cycle 5 (CDC5) is associated with miRNA which is a positive transcription factor. The drosophila exportin-5 ortholog HASTY (HST) factor exports miRNA duplex to the cytoplasm.

The miRNA-targeted genes may be solitary members of a gene group or synchronize various family groups. However, more than one type of miRNA gene can regulate a single individual with tissues and a single-miRNA gene can control many family individuals. The territorial and worldly articulation of plenitude-developed miRNAs is firmly managed; they differ enormously within various miRNAs, and the bounty likewise fluctuates relying upon the type of tissues or growth and embryonic stages [16].

The miRNAs inhibit the activity of mRNA to control the gene expression at the level of post-transcription. In contrast to animals, plants have an ideal relationship between mRNA and miRNA [17]. For the silencing, a ribonucleoprotein RNA-induced silencing complex (RISC) is formed [18]. Different types of slicers have been reported: AGO10, AGO7, AGO4, AGO2, and AGO1. However, AGO1 is mostly related to miRNA [19]. The RNA splitting is activated by AGO1 by inhibiting specific genes [20].

**Figure 1:**
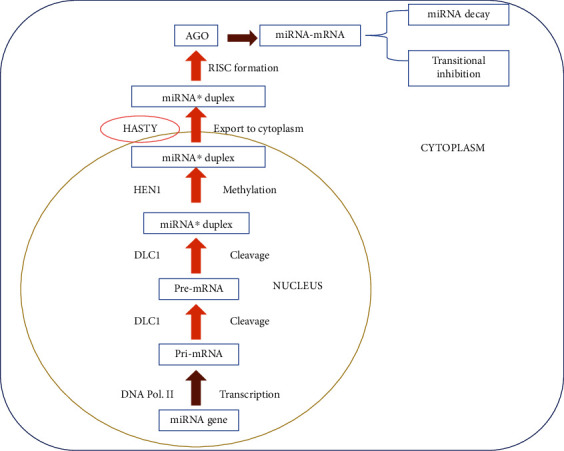
Mechanism of action of miRNAs in plants. miRNA: microRNA; pri-miRNA: primary transcripts; DCL1: RNAase dicer-like 1.

In plants, the genes coding for transcription, stress response proteins, and other factors which are associated with different biological mechanisms are controlled by miRNA-like conservation of genetic materials, maintenance of metabolism, and enlargement of plants, signal transduction, energy pathway, homeostasis, natural immunity, and adaptive feedback to biotic and abiotic stress [21, 22]. The phytochemicals are peripheral metabolites that play an important role in different processes which are related to the association between plants and its surrounding [23–25]. The mechanism of action of miRNAs in plants is shown in Figure 1.

These compounds involve isoprenoids, tannins, alkaloids, phenols, glycosides, and triterpene glycosides, which protect the plants from different living and nonliving stress-causing factors [26, 27]. Such kinds of molecules are produced by herbs for self-protection; these compounds are also used in various industries like nutraceuticals, flavouring F compounds, dyes, and insecticides as they are providing a great response in terms of human health [28, 29]. Materialistic significance has brought about an extraordinary enthusiasm for considering the prospects of improving its creation [30]. It concluded that miRNAs regulate several biological cycles at the stage of post-transcription. Several recent investigations concluded that the miRNA plays an important part in the transduction of the peripheral metabolic series [31]. So, the products of peripheral metabolites can be regulated by miRNAs. The production of any metabolite can be regulated by a positive or negative feedback mechanism, so we can stop the production of undesired products and enhance the production of desired metabolites [32].

Computational evaluation executed in two transcriptomes of Swertia ensured to identify miRNAs connected with secondary metabolites, miR-11320, miR-168, miR-156a, miR-11071, miR-166a, and miR-166b focused on metabolic enzymes consisting of premnaspirodiene oxygenase, aspartate aminotransferase, phosphoglycerate mutase, ribulose-phosphate 3-epimerase, acetyl-CoA, and acetyltransferase. Additionally, it consists of a gene encrypting a homeobox-leucine zipper protein (HD-ZIP) along with secondary metabolite organic production in *Swertia chirayita* (Roxb.) Buch.-Ham. ex C.B.Clarke [33].

The production of self-protecting metabolites is reduced in diseased plants due to alteration in gene expression so these may be maintained by miRNAs. When *Solanum tuberosum* L. is exposed to sunlight, brilliance-reactive miRNAs are produced which plays an important role in nicotine metabolism, UMP salvage, fat production, and cellulose catabolism [34].

The stress of cadmium in oil seed rape (*Brassica napus* L.) described miRNA as a crucial element in the synchronization of transcription factors, living thing stress protection, ion balancing, and peripheral metabolism production [35].

*Nicotiana tabacum* tainted with tobacco mosaic virus (TMV), at the beginning phase of the disease (5 dpi), shows a bunch of miRNAs with low aggregation, while a large portion of the miRNAs was upregulated at 15 and 22 dpi including both miRNAs and miRNA targets [36, 37].

## 3. Flavonoid Biosynthesis and miRNA

Flavonoids have a hydroxylated phenolic structure and contain benzo-*γ*-pyron, which is obtained from phenylpropanoid [38, 39]. Flavonoids have different compounds including chalcones, catechins, flavonols, flavonols, anthocyanins, flavanones, and flavanonols. These compounds are synthesized with the help of others in plants and microorganisms, and their concentration varies as per the environmental conditions [40, 41].

The biological functions of flavonoids vary greatly with the structure and result in various bioactivities such as antimutagenic, antioxidative, anticarcinogenic, anti-inflammatory, and antiplatelet aggregation [42]. These biological activities of flavonoids impart different applications such as medicinal, nutraceutical, cosmetics, and pharmaceutical [43–45].

miRNA-initiated reactions take part in the assemblage of peripheral metabolites (Table 1).

Scarce studies are present about the importance of miRNAs in the organic synthesis of flavonoids. In *Helianthus*, 323,318 ESTs had been logically observed for the miRNA recognition and the miR911 group was observed being connected to the biosynthesis of tocopherols. Himalayan May apple (*Podophyllum hexandrum* Royle), miR1438, affects caffeoyl-CoA O-methyltransferase and is associated with flavonoid biosynthesis; phenylalanine metabolism; stilbenoid, diarylheptanoid, and gingerol biosynthesis; and phenylpropanoid biosynthesis.

**Figure 2:**
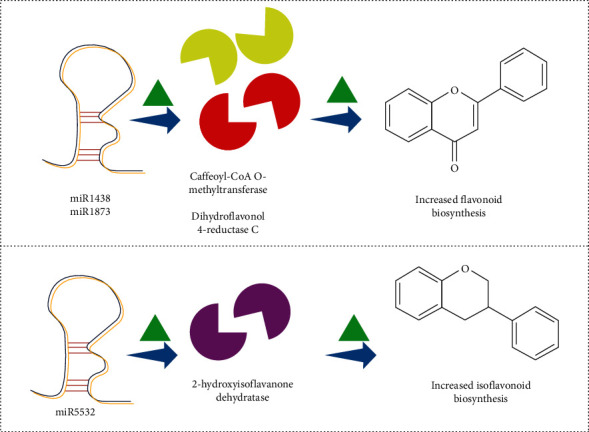
miRNA increases the activity of biosynthetic enzymes involved in flavonoid and isoflavonoid production and resulting in an improved yield of flavonoid and isoflavonoids.

Dihydroflavonol 4-reductase C is affected with miR1873 which is associated with flavonoid biosynthesis (Figure 2). Isoflavonoid biosynthesis is associated with the miR5532 2-hydroxyisoflavanone dehydratase (Figure 2; Table 1) [56].

miR2911 regulates tocopherol synthesis genes such as gamma-tocopherol methyltransferase in sunflower plants (*Helianthus annuus* L.) [46]. Furthermore, mRNA (miR828a and miR948a) helps to accelerate flavonoid biosynthesis by regulating *MYB12* lipoxygenase in Clary [47]. Flavonoid's synthesis (curcumin) in turmeric plants (*Curcuma longa* L.), miR2919, miR1168.2, miR156b, and miR1858 promote the flavanone synthase gene [48].

Anthocyanins are one of the important members of the flavonoid family [67, 68]. In the tuberous root of sweet potato, the expression of ib-miR156 is its target ibSPL, which aids in anthocyanin production [49]. Kiwifruit, miR858, reported accelerating anthocyanin accumulation [69]. High microR156 movement initiates aggregation of anthocyanin along with movement initiated by flavonols. The current study also illustrated that squamosa promoter-binding-like protein 9 SPL9 adversely initiated anthocyanin aggregation with the disruption of an MYB-bHLH-WD40 transcriptional-initiated system. *Diospyros kaki* L.f. fruit results acquired at tested periods (15 and 20 WAF) confirmed the contrasting appearance of the messenger RNAs, suggesting that those miRNAs throughout the growth and a number of them are miRNAs 858 and 56 which adjust the synthesis of PA. PA production genes are positively initiated, miR858 but negative effects are observed by miR156. Another miR395 and miR858b play their role in biosynthesis proanthocyanidin [51]. Some miRNAs (U4351355, U3938865, U805963, U977315, and U436803) initiate lipid and flavonol organic production in *Lonicera japonica* Thunb. [2, 3]. Yang et al. [52] imply that salty situations modify miRNAs in which few salt pressure-associated organic channels consist of flavonoid biosynthesis, calcium signaling pathway, and plant hormone signal transduction [52].

Gou and colleagues reported that in the stem of *Arabidopsis thaliana* anthocyanins accumulate in an acropetal manner, this model is controlled by SPL (squamosa binding protein-like) genes and stimulated by miR156 [53]. The depiction of *A. thaliana* indicates that miR858a targets myeloblastosis (MYB) transcript elements which are engaged in flavonoid organic production, development, and growth. MYB transcription factors down synchronize with overexpression of miR858a and the redirection of metabolic flux initiated with the excessive appearance of MYBs concerning the composition of flavonoids [54]. The biosynthesis of flavonoids (anthocyanins) is regulated by miR156, miR858a by stimulating the transcription factors R2R3-MYB and SPL9 [53, 54]. *Rauvolfia serpentine* (Indian snakeroot) has two essential types of miRNA such as miR396b and miR828a, both of which targets kaempferol 3-O-*β*-D-galactosyltransferase and anthocyanin regulatory C1 protein for synthesizing flavonol glycoside and anthocyanin biosynthesis, respectively [55].

*Podophyllum hexandrum* Royle (May apple) is an endangered medicinal plant [56]. The Kyoto Encyclopedia of Genes and Genomes (KEGG) pathway study demonstrated that miR1438 and miR1873 regulate various metabolic pathways, especially the biosynthesis of secondary metabolites like lignin and flavonoid via caffeoyl-CoA *O*-methyl transferase and dihydroflavonol 4-reductase C gene, respectively [56]. Biswas et al. [56] further revealed that miR5532 upregulates the 2-hydroxyisoflavanone dehydratase gene for synthesizing isoflavonoid in the May apple tree. Flavonoid biosynthesis also occurred in May apple through inducing chalcone synthase and 4-coumarate–CoA ligase. Both of them are targeted by miR829.1 and miR172i, respectively [56]. 4-Coumarate-CoA ligase catalyzes the activation of the corresponding thiol esters by 4-coumarate and other 4-hydroxycinnamates. These activated thiol esters are subsequently employed as flavonoid and monolignol precursors [70]. miR5015 has been shown to influence the production of gingerol by suppressing phenyl ammonia-lyase (PAL) which is a basic enzyme in the synthesis [57]. *Glycine max* (L.) Merr. (soybean) CHS-siRNA targets chalcone synthase enzyme to the biosynthesis of flavonoids [58, 59].

In *Pyrus bretschneideri* Rehder, miR1061-3p regulates naringenin 3-dioxygenase, which produces flavonoids [60]. *Helianthus annuus* produces *α*-tocopherol, and flavones by miRNA and miRNA influence the regulation process via targeting *γ*-tocopherol methyltransferase, isoflavone 20-hydroxylase, and dihydroflavonol 4-reductase [2]. miR156, miR828, and miR858 are reported to play an important function in regulating anthocyanin biosynthesis in radish through MYBs, bHLH, WD40, SPLs, ARF, EIN3, WRKY, MADS-box, sucrose synthase, sugar/inositol transporter, and ABC transporter genes [61]. Furthermore, MYB114 and flavonoid 3′-monooxygenase (F3MO) are also targeted by miR828 and miR858 in the grape plant for the biosynthesis of flavonol [62]. MYB, an essential step in flavonol biosynthesis, upregulates CHI, CHS, and FLS genes for the production of flavonoids [60, 63, 71].

Sun and coworkers reported that the expression of eight miRNAs (csn-miR160a, csn-miR396a, csn-miR167a, csn-miR4380a, csn-miR3444b, csn-miR5251, csn-miR7777-5P.1, and miR2593e) and their target genes (auxin response factor 18, growth-regulating factor 7, calcium-transporting ATPase 13, DNA-directed RNA polymerase V subunit, auxin response factor 6 ARF6, CHI enzyme, DFR, C4H, and ANR) is involved in catechin biosynthesis [64]. By decreasing the expression of their biosynthesis-related gene transcription, miRNAs could adversely control catechin production.

Another study reported that miR166, miR169, miR398 target HD-ZIP, NF-YA, and GATA gene in the tea plant [65] as well as miR6194 influence flavanone 3*β*-hydroxylase (F3H) for flavonols and anthocyanidin biosynthesis [52].

## 4. Terpenoid Biosynthesis and miRNA

Isopentenyl diphosphate (IPP) and dimethylallyl diphosphate (DMAPP) are a C5 predecessor of plant terpenoid secondary metabolites. Terpenoids range in different groups according to the orders of carbon atoms such as polyphenols (>45), carotenoids (C40), triterpenes (C30), diterpenes (C20), sesquiterpenes (C15), and monoterpenes (C10) [72]. Terpenoids also have different applications as biological and industrial issues like flavonoids and alkaloids. The computational study identifies the change of miRNAs into 6 transcriptomes of *Picrorhiza kurroa Royle* ex Benth. which discovered that miRNA-4995 plays a promoter function in the biosynthesis of terpenoids (Table 2), finally disturbing the manufacturing of picroside-I [73]. The miR-5021 effect on two kinds of enzymes that take part in organic synthesis of GCPE protein, indole alkaloids (IAs), and terpenoid cyclases was first time observed in *Catharanthus roseus* (L.) G.Don [74]. miRNAs (1134, 5021, and 7539) are probably concerned with promoting terpenoid organic synthesis through the terpenoid channel genes; nontranscriptional factor proteins, like IDS, DXS, IDI, and HMGR, are necessary to bring out DMAPP and IPP, the main predecessor for all kinds of downstream terpenoids [75].

miRNAs (6435, 6449, 7540, 5491, 5183, and 5255) affect downward enzymes in the organic synthesis of mono-, sesqui-, di-, and tri-terpenoids; they included germacrene A oxidase, R-linalool synthase, beta-amyrin synthase (bAS), gibberellin 3-oxidase, squalene epoxidase, and ent-kaurene synthase [75]. miRNAs (5658, 5251, 5021, 2919, and 838) were recognized to be included in the terpene organic synthesis channel in *Ferula gummosa* Boiss.

Squamosa promoter binding and ATHB13 were observed to be promoted by miRNAs (1533, 5021, and 5658) [76]. Sesquiterpene biosynthesis is mediated by miRNA156-connected SPL gene [77]. miR414 is involved in the triterpenoid and sesquiterpenoid organic syntheses while miR5021 also plays a role in terpenoid formation (Table 1) [66]. Investigators revealed that SPL9 specifically connects to and triggers the TPS21 promoter. Due to the great conservation of miR156-SPLs in plants, the findings establish a molecular connection between the developmental time and sesquiterpene synthesis and provide a novel method for engineering plants to grow faster while producing more terpenoids [77]. In all, 130 unique candidate transcripts for eight miRNA families were anticipated. All anticipated miRNA targets govern development, reproduction, stimulus response, signaling, and various metabolic processes. Additionally, a network of miRNA-mediated gene regulation was built using the hybridized lowest free energy of discovered miRNAs and their targets. The study discovered that the mint family's miR414, miR156, and miR5021 might regulate the essential oil biosynthesis gene control mechanism. Additionally, three miRNA candidates were identified as important in trichome formation (miR5021, miR156, and miR5015b) [66]. However, apart from providing first-hand knowledge on *Rauvolfia serpentina* (L.) Benth. ex Kurz miRNAs and their objectives, the outcomes in this work help to a profound comprehension of miRNA-mediated gene regulation mechanisms in plants [55].

Another study demonstrated that among 452 recognized miRNAs, which equate to 589 precursor miRNAs, 62 miRNAs are expressed exclusively in the base, 95 miRNAs are expressed exclusively in the stem, 19 miRNAs are expressed exclusively in the leaf, and 71 miRNAs are expressed exclusively in flower. The degradome study found 69 targets that may be cleaved by 25 miRNAs. Among these, miR5072 cleaved acetyl-CoA C-acetyltransferase, which is implicated in the production of tanshinones. This investigation expanded significantly to our comprehension of the tissue-specific expression patterns of miRNAs in *Salvia miltiorrhiza* Bunge and laid the groundwork for future exploration on the production of tanshinone mediated by miRNAs [78].

Moreover, recent findings indicated that most anticipated miRNAs were engaged in rhizome formation control. miR854, miR5021, and miR838 have been revealed as miRNAs that control ginger rhizome growth and essential oil production [57]. It is demonstrated that miRNA-4995 performs a key role in regulating terpenoid biosynthesis, subsequently influencing the development of picroside-I. miR-5532 and miR-5368 expression rates were significantly lower in field-grown specimens than *in vitro*-cultured samples, implying a role in controlling *Picrorhiza kurroa* Royle ex Benth. development under culture environments [73]. Additionally, an investigation concluded 60 mature miRNAs and 6 precursor miRNAs in *Podophyllum hexandrum* Royle using 454 pyrosequencing. The finding contributes fundamental knowledge regarding the control of secondary metabolite production in *Podophyllum hexandrum* Royle by miRNAs [56].

A study demonstrated to pinpoint the trichome-specific miRNAs and mRNA targets. The findings from the experiment give a basis for additional research in *Xanthium strumarium* L. glandular cells to elucidate the governing strategy of miRNAs underpinning the production of secondary, in particular, terpenoids [75]. Researchers discovered that a large number of the miRNAs pinpointed had tissue-specific expression. By scanning the ginseng EST database for anticipated objectives of the estimated 69 conserved miRNAs, 346 promising targets were recognized. The anticipated targets were primarily engaged in secondary metabolic processes responding to transcription regulator activities and biotic and abiotic stress, among other metabolic pathways [79]. Conversely, miR390 was expected to engage a gene associated with trichome formation, the location of artemisinin production, and therefore may be possible for genetic transformation to increase the artemisinin content [72]. Also, a new study demonstrated insight into the role of miRNAs in the control of secondary metabolite production in the herbal plant *Swertia chirayita* (Roxb.) Buch.-Ham. ex C.B.Clarke. Additionally, the putatively discovered miRNAs can increase secondary metabolite production in *S. chirayita* by genetic modification [33].

**Figure 3:**
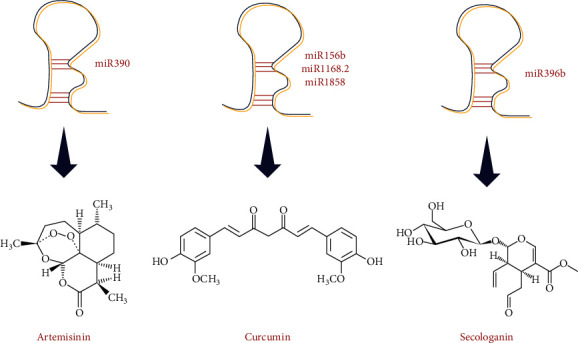
miRNA improves the production of terpenoid compounds.

Another study revealed that three miRNAs (miR156b, miR1168.2, and miR1858) were involved in curcumin production. Other miRNAs were discovered to be engaged in turmeric's development and growth. Additionally, a phylogenetic analysis of selected miRNAs was done in this study [48]. Moreover, another investigation demonstrated the identification of miRNA associated with control gene channels regulating terpenoid biosynthesis in *Camellia sinensis* (L.) Kuntze at various growth stages [65]. Investigating the regulation of terpenoid biosynthesis by miRNA outcomes indicates that six miRNAs examined control terpenoid production in *P. minor* post-transcriptionally. This governmental function of miRNAs suggests that they may be used as a genetic tool to control terpenoid production in *Persicaria minor* (Huds.) Opiz [80]. Another study also evaluated miRNAs and their presumed target genes involved in transthyretin-like (TTL) biosynthesis, and the results showed the existence of a complex miRNA-mRNA regulatory network involved in *Ginkgo biloba* L. TTL metabolism [81].  Figure 3 shows few examples in which miRNA assists in the improved production of terpenoid compounds.

The investigation uncovered multiple previously unknown miRNA families in *Artemisia annua* L., including miR399, miR396, miR319 miR858, miR6111, and miR5083. The expression patterns and correlations between miRNAs and their associated targets in *A. annua* plant are different, depending on its developmental phases [82]. Saifi and coworkers found nine miRNAs (miR319c, miR319a, miR319f, miR319b, miR319h, miR319d, miRstv-7, miR319e, and miRstv-9) and authors reported that these miRNAs are correspondingly connected to the expression thresholds of their target mRNAs and steviol glycosides were detected to be directly connected to the expression stages of their target mRNAs. This work established a foundation for a better knowledge of the steviol glycoside biosynthetic process, and these miRNAs may be used to control the production of these metabolites in *Stevia rebaudiana* (Bertoni) Bertoni to increase their concentration and productivity [83].

## 5. Alkaloid and Other N-Containing Metabolites and miRNA

Alkaloids are naturally occurring organic compounds that contain one or more nitrogen atoms in their heterocyclic ring structure, have alkali-like properties [84], have a variety of biological effects such as antimalarial (e.g., quinine), antiasthma (e.g., ephedrine), anticancer (e.g., homoharringtonine) [85], cholinomimetic (e.g., galantamine) [86], vasodilatory (e.g., vincamine), antiarrhythmic (e.g., quinidine), analgesic (e.g., morphine) [87], antibacterial (e.g., chelerythrine) [88], and antihyperglycemic activities (e.g., piperine) [63], and play significant roles in the defence against herbivores [89].

In plants, a myriad of bioactive compounds is synthesized naturally by several pathways. Among them, the involvement of miRNA is the most common way used for the biosynthesis of naturally occurring alkaloids. Pyrrolidine, purine, imidazole, indole, quinolizidine, pyrrolizidine, isoquinoline, and tropane are among the various types of alkaloids listed [90]. Disparate to other bioactive compounds, this group is extremely diverse and heterogeneous with an estimated 12,000 alkaloids in nature [89]. Alkaloids according to their toxic nature react as resistance compounds against many kinds of herbivores and pathogens. The understanding of alkaloid biosynthesis balancing is critical for its production.

Tobacco (*Nicotiana tabacum* L.) belongs to the Solanaceae family; a study revealed six different kinds of distinctive tobacco miRNAs, namely, miRX13, miRX17, miRX19, miRX20, miRX27 [50], and miR164 [91], that have been anticipated to target important genes of the nicotine organic biosynthesis and dissimilation channel, cytochrome P450 monooxygenase (CYP82E4), quinolinate phosphoribosyl-transferase 1 (QPT1), quinolinate phosphoribosyl-transferase 2 (QPT2), putrescine methyltransferase 2 (PMT2), and NtNAC-R1 genes [50].

**Figure 4:**
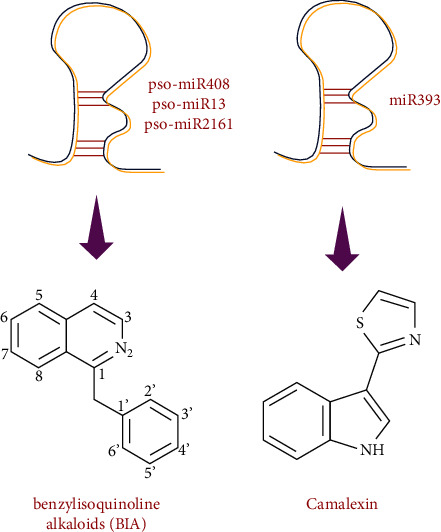
Role of miRNA in the synthesis of alkaloid and other N-containing metabolites.

In the opium poppy, the botanical name of *Papaver somniferum* L., a member of the Papaveraceae family, it has been identified that transcription of pso-miR408, pso-miR13 and pso-miR2161 (Table 1) has an important role in the biosynthesis of organic benzylisoquinoline alkaloids (BIA) [92]. pso-miR13, pso-miR2161, and pso-miR408 would possibly break 7-O-methyltransferase, S-adenosyl-l-methionine:3′-hydroxy-N-methylcoclaurine, 4′-O-methyltransferase 2/4′-O-methyltransferase 2 (4-OMT)/FAD-binding, and BBE domain-containing protein, respectively, transcript concerned with inside the transformation of S-reticuline to morphinan alkaloids (BIA). 4-OMT is the target of pso-miR216 and moderates the assembly of S-reticuline; this is additionally an intermediary particle in BIA organic production. On the opposite side, pso-microR408 feasibly reacts with messenger RNA from reticuline oxidase-like protein in the rate of transformation of S-reticuline to (S)-scoulerine within the BIA channel [92].  Figure 4 showed the role of miRNA in the synthesis of alkaloid and other N-containing metabolites.

Two kinds of two paclitaxel biosynthetic genes in *Taxus baccata* L., namely, taxane 2-*α*-O-benzoyl transferase and taxane 13-*α*-hydroxylase, are the breaking objects of microR164 and 171 [93, 94]. *In silico* inspection identified that microR396b in *Rauvolfia serpentina* (L.) Benth. ex Kurz hits kaempferol 3-O-beta-D-galactosyltransferase whose interest as transferase shifted the hexosyl class for the development of flavonoid glycosides [55]. miR-5021 in Madagascar periwinkle (*Catharanthus roseus* (L.) G.Don) regulates the enzyme involved in the biosynthesis of TIAs which includes UDP-glucose iridoid glucosyltransferase [74].

In Madagascar periwinkle, there are some other miRNAs such as cro-miR160, cro-miR164a, cro-miR164b, and cro-miR393d, which have a regulatory role in targeting CrARF16, *ORCA3*, *ORCA4*, and *BIS1* genes in the TIA biosynthetic pathway and synthesis indole alkaloids [95]. In addition, Robert-Seilaniantz et al. [96] demonstrated that miR393 with its related auxin receptor genes is involved in alkaloid (camalexin) biosynthesis in Thale cress (*Arabidopsis thaliana* (L.) Heynh.) and in turmeric also named as *Curcuma longa*; miR5021 is illustrated to have a role to synthesize isoquinoline alkaloid interacting with two enzyme genes like aspartate transaminase and aromatic-amino-acid transaminase [48]. Furthermore, in sunflower plants, U3938865 and U4351355 involved glucosinolates and camalexin (indole alkaloid) biosynthesis through regulating the MYB34 gene [2] (Table 3).

## 6. Fatty Acids and Other Compound Biosynthesis and miRNA

miRNA plays an important role in fatty acid secondary metabolite biosynthesis [97]. *Lonicera japonica* targets regulatory genes which help in the process of synthesis such as acyl-CoA synthetase, acyl carrier protein, and fatty acid hydroxylase. miRNAs involved in the fatty acid synthesis are U436803, U977315, and U805963 [2]. Besides this, in *Paeonia × suffruticosa* Andrews (tree peony) plant, fatty acids are synthesized by miR156b and miR7826 via regulating 1-acyl-sn-glycerol-3-phosphate acyltransferase and ACCase gene activities [98].

In *Corynespora cassiicola* plant, some novel miRNA was found that positively or negatively regulates secondary metabolite production in plants. These novel types of miRNAs are novel-miR1 to novel-miR7 which targets genes in phenylpropanoid synthesis [15]. Furthermore, phenylpropanoid biosynthesis is also regulated by miRNA (miR2673a and miR396b) in *Podophyllum hexandrum* Royle [99].

Withanolide is a class of active secondary metabolites in plants with medicinal interest. miR5140, miR159, miR477, and miR530 upregulated cycloartenol synthase (CAS1) sterol delta-7 reductase 1, CYP82G, zeatin o-glycosyl transferase (UGTs), and secoisolariciresinol dehydrogenase (ABA2) for synthesizing withanolide in ashwagandha plant [100]. Moreover, the tobacco plant synthesized glucosinolate with the help of miRNAs [53]. In *Arabidopsis thaliana* (L.) Heynh., miR5090 and miR826 proportion target AOP2 encrypting a 2-oxoglutarate-structured-dioxygenase, which is concerned in sulfur and nitrogen organic production [49, 101].

miRNAs had been observed from laboratory subculture of plant essential sections of the transcriptome of *Withania somnifera* (L.) Dunal in which miRNAs 5303, 159, 5140, and 172 are found in root tissues and miRNAs 5079, 530, 477, and 1426 are found in leaf tissues. Such miRNA is connected to the elongation of peripheral metabolites. Endogenetic mRNAs from roots (159 and 5140) and leaves (477 and 530) may be involved in the increase of metabolite production, although miR530 from leaves and miR172 and miR159 from roots were entangled in the balancing of peripheral metabolites connected with mRNAs [100]. *Arabidopsis thaliana* (L.) Heynh., *Oryza sativa* L., and *Chlorophytum borivilianum* Santapau & R.R.Fern.-targeted gene forecast imply that miR166, miR172, miR894, and miR9662 are probably concerned in enhancing the organic production of saponin [102]. miRNAs (5298b and 8154) raise phenylpropanoid, taxol, and flavonol organic synthesis in subcultured Taxus cells [103]. *Salvia miltiorrhiza* Bunge miRNA5072 targets acetyl-CoA which is concerned with the organic synthesis of tanshinone [78] (Table 4). miRNA826 targets hydroxyalkyl generating 2 oxoglutarate dioxygenases that is concerned with sulfur and nitrogen production [101].

## 7. Conclusion

miRNAs are microscopic particles related to growing functions that direct gene expression. The method assumed post-transcriptional and transductional procedures. miRNA secondary metabolism dominance is the latest kind of field and better information about the process of peripheral metabolism in plants will play a critical role in achieving new kinds of outcomes in controlled systems. These outcome by-products have good economic values due to their utilization in cosmetics product, food, agronomy, and other industries. Further investigations for the production of secondary metabolites by maneuvering the role of miRNA in various other crops such as spices, flowering plants, and medicinal plants may result in improving the profitability of the food and pharmaceutical industries.

## Figures and Tables

**Table 1:** miRNA effect on the synthesis of secondary metabolites.

Botanical name	Common name	Family	miRNA	Target	Function	References
*Helianthus annuus* L.	Sunflower	Asteraceae	miR2911	Gamma-tocopherol methyltransferase	Tocopherol biosynthesis	[46]
*Salvia sclarea* L.	Clary	Lamiaceae	miR828a, miR948a	MYB12 lipoxygenase	Flavonoid biosynthesis	[47]
*Curcuma longa* L.	Turmeric	Zingiberaceae	miR2919	Flavanone synthase		[48]
			miR1168.2, miR156b		Curcumin biosynthesis	
			miR1858			
*Ipomoea batatas* (L.) Lam.	Sweet potato	Convolvulaceae	ib-miR156	ibSPL	Anthocyanin biosynthesis	[49]
*Actinidia arguta* (Siebold & Zucc.) Planch. ex Miq.	Kiwifruit	Actinidiaceae	miR858	AaF3H, AaLDOX		[50]
				AaF3GT		
*Diospyros kaki* L. f.	Kaki	Ebenaceae	miR395p-3p	bHLH, MYB	Proanthocyanidin biosynthesis	[51]
			miR858b			
*Lonicera japonica* Thunb.	Japanese honeysuckle	Caprifoliaceae	miRNAs (U436803, U977315, U805963, U3938865, 4351355)	R2R3-MYB transcription factors	Flavonoid biosynthesis	[2, 3]
*Halostachys caspica* C.A.Mey.	Bail	Amaranthaceae	miR6194	Flavanone 3-hydroxylase	Flavonol, anthocyanidin, and proanthocyanidin syntheses	[52]
			miR5655			
*Arabidopsis thaliana* (L.) Heynh.	Thale cress	Brassicaceae	miR156^∗^	SPL9	Anthocyanin biosynthesis	[53]
			miR858a^∗^	R2R3-MYB transcription factors	Flavonoid biosynthesis	[54]
			miR156	SPL transcription factor, transcription factors	Flavonoid biosynthesis	[53, 54]
			miRNA858a			
			R2R3-MYB			
*Rauvolfia serpentina* (L.) Benth. ex Kurz	Indian snakeroot	Apocynaceae	miR396b	Kaempferol 3-O-beta-dgalactosyltransferase	Flavonol glycoside	[55]
			miR828a	Anthocyanin regulatory C1 protein	Anthocyanin biosynthesis	
*Podophyllum hexandrum* Royle	May apple	Berberidaceae	miR1438	Caffeoyl-CoA O-methyl transferase	Lignin biosynthesis	[56]
			miR1873	Dihydroflavonol 4-reductase C	Flavonoid biosynthesis	
			miR1873	Phenylalanine ammonia lyase (PAL)	Gingerol (phenolic) biosynthesis	[57]
			miR5532	2-Hydroxyisoflavanone dehydratase	Isoflavonoid biosynthesis	[56]
			miR1873/miR5532	Dihydroflavonol	Flavonoid/isoflavonoid biosynthesis	
				4-Reductase C/-hydroxyisoflavanone dehydratase		
			miR172i	4-Coumarate–CoA ligase	Flavonoid biosynthesis	
			miR829.1	Chalcone synthase		
*Glycine max* (L.) Merr.	Soybean	Fabaceae	CHS-siRNA	Chalcone synthase	Flavonoid biosynthesis	[58, 59]
*Pyrus bretschneideri* Rehder	Pear tree	Rosaceae	miR1061-3p	Naringenin 3-dioxygenase	Flavonoid biosynthesis	[60]
*Helianthus annuus* L.	Sunflower	Asteraceae	miR2911	*γ*-Tocopherol methyl transferase	*α*-Tocopherol biosynthesis	[46]
			miRNAs (U4992168, U2743257)	Isoflavone 20-hydroxylase; dihydroflavonol 4-reductase	Flavones biosynthesis	[2]
*Raphanus sativus* L.	Radish	Brassicaceae	miR156	MYBs, bHLH, WD40, SPLs, ARF, EIN3, WRKY, MADS-box, sucrose synthase, sugar/inositol transporter ABC transporter	Anthocyanin biosynthesis	[61]
			miR828			
			miR858			
*Vitis vinifera* L.	Grape	Vitaceae	miR828	MYB114, flavonoid	Flavonol biosynthesis	[62]
			miR858	3′-Monooxygenase (F3MO)		
*Osmanthus fragrans* Lour.	Sweet osmanthus	Oleaceae	miR858, miR858a	MYB1*- >* CHI, CHS, FLS	Flavonoid synthesis	[63]
*Camellia sinensis* (L.) Kuntze	Tea	Theaceae	csn-miR160a	Auxin response factor 18	Catechin biosynthesis	[64]
			csn-miR396a	Growth-regulating factor 7, calcium-transporting ATPase 13, DNA-directed RNA polymerase V subunit		
			csn-miR167a	Auxin response factor 6 ARF6, CHI enzyme		
			csn-miR4380a	DFR		
			csn-miR3444b			
			csn-miR5251	C4H		
			csn-miR7777-5P.1			
			miR2593e	ANR		
			miR166, miR169	HD-ZIP, NF-YA, GATA	Phytohormone biosynthesis (gallated catechin, caffeine, theanine)	[65]
			miR398			
*Mentha* spp.	Mint	Lamiaceae	miR156	Basic helix-loop-helix (bHLH)	Flavone/flavonol biosynthesis	[66]
*Halostachys caspica* C.A.Mey.	Yan Sui Mu	Berberidaceae	miR6194	Flavanone 3b-hydroxylase (F3H)	Flavonols, anthocyanidins	[52]
					Biosynthesis	

**Table 2:** MicroRNA effect on synthesis of terpenoid secondary metabolites.

Botanical name	Common name	Family	miRNA	Target	Function	References
*Picrorhiza kurroa* Royle ex Benth.	Kutki	Scrophulariaceae	iRNA-4995	3-Deoxy-7-phosphoheptulonate synthase (DAHP synthase)	Terpenoid biosynthesis	[73]
*Catharanthus roseus* (L.) G.Don	Tropical periwinkle	Apocynaceae	mir-5021	MYB transcription factor, geranyl diphosphate synthase, GCPE protein, UDP-glucose iridoid glucosyltransferase	Isoprenoid/terpenoid biosynthesis	[74]
*Ferula gummosa* Boiss.	Galbanum	Apiaceae	miR2919, miR5251, miR838, miR5021, miR5658	SPL7, SPL11, ATHB13 TFs	Terpene biosynthesis	[76]
*Pogostemon cablin* (Blanco) Benth.	Patchouli	Lamiaceae	miR156^∗^	SPL9	Sesquiterpenoid, triterpenoid biosynthesis	[77]
*Mentha* spp.	Mint	Lamiaceae	miR156, miR414, and miR5021	Basic helix-loop-helix (bHLH) geranyl di-phosphate synthase subunit alpha-like protein (NACA)	Terpenoid backbone biosynthesis	[66]
			miR156^∗^	1-Deoxy-D-xylulose	Terpenoid biosynthesis	[66]
				5-Phosphate synthase (DXS)		
			miR414	Terpene synthase 21 (TPS21)	Sesquiterpenoid, triterpenoid biosynthesis	
*Rauvolfia serpentina* (L.) Benth. ex Kurz	Indian snakeroot	Apocynaceae	miR396b	Secologanin synthase	Secologanin	[55]
*Salvia miltiorrhiza*	Red sage	Lamiaceae	miR5072	Acetyl-CoA C-acetyl transferase	Tanshinones (abietane-type norditerpenoid quinones)	[78]
*Zingiber officinale* Roscoe	Ginger	Zingiberaceae	miR838	CYP71	Terpenoid metabolism	[57]
*Picrorhiza kurroa* Royle	Kutki	Plantaginaceae	miR4995	3-Deoxy-7-phosphoheptulonate synthase (DAHP synthase)	Picroside biosynthesis	[73]
*Podophyllum hexandrum* Royle	Mayapple	Berberidaceae	miR5538	Protein-S-isoprenylcysteine	Terpenoid backbone biosynthesis	[56]
				O-methyltransferase		
*Xanthium strumarium* L.	Rough cocklebur	Asteraceae	miR7539, miR5021, miR1134	Upstream genes of terpenoid pathway	Terpenoid	[75]
			miR5491, miR5255	Beta-amyrin synthase	Xanthanolide (sesquiterpenoids)	
			miR6449, miR5183	Squalene epoxidase		
			miR7540	Ent-kaurene synthase		
				Gibberellin 3-oxidase		
				R-Linalool synthase		
			miR7540, mmiR5183, miR6449, miR5255, miR5491, miR6435	R-Linalool synthase, gibberellin 3-oxidase, ent-kaurene synthase, squalene epoxidase, beta-amyrin synthase, germacrene A oxidase	Mono-, sesqui-, di-, and triterpenoid biosynthesis	
			miR1134	3-Hydroxy-3-methylglutaryl coenzyme A reductase (HMGR)	Terpenoid backbone biosynthesis	
			miR5183	Gibberellin 3-oxidase	Diterpenoid	
			miR5255	Squalene epoxidase	Triterpenoid	
			miR5491	Beta-amyrin synthase	Triterpenoid	
			miR6435	Germacrene A oxidase	Sesquiterpenoid	
			miR6449	Ent-kaurene synthase	Diterpenoid	
			miR7539	1-Deoxy-D-xylulose	Terpenoid backbone	
				5-Phosphate synthase (DXS)		
			miR7540	R-Linalool synthase	Monoterpenoid	
*Panax ginseng* C.A.Mey.	Asian ginseng	Araliaceae	miR854e miR854b miR854c	Farnesyl diphosphate synthase, squalene epoxidase (SE)	Ginsenosides (triterpene)	[79]
			miR1439b, miR1439h	Beta amyrin synthase		
*Artemisia annua* Pall.	Sweet wormwood	Asteraceae	miR390	A gene involved in trichome development	Artemisinin (sesquiterpene)	[72]
*Swertia chirayita* (Roxb.) Buch.-Ham. ex C.B.Clarke	Swertia	Gentianaceae	miR-168	Acetyl-CoA acetyltransferase	PSMs	[33]
			miR-11320	Aspartate aminotransferase		
			miR-166a	Premnaspirodiene oxygenase		
			miR-11071	Ribulose-phosphate3-epimerase		
			miR-156a	Phosphoglycerate mutase,		
			miR-166b			
*Curcuma longa* L.	Turmeric	Zingiberaceae	miR5649a	Geranylgeranyl-diphosphate specific	Terpenoid backbone biosynthesis	[48]
			miR5021	Geranyl-diphosphate synthase		
				1-Deoxy-D-xylulose-5-phosphate synthase (DXS)		
				Geranylgeranyl-diphosphate synthase		
*Camellia sinensis* (L.) Kuntze	Tea	Theaceae	miR3630	*MYC2*	Terpenoid biosynthesis	[65]
			miR33418	*MYB.*		
			miR156f	*SPL9*		
			miR156	AaERF1/2		
			miR535			
			miR858			
*Persicaria minor* (Huds.) Opiz	Kesum	Polygonaceae	pmi-miR530 pmimiR6173	MVD, probable sulphate transporter; Sesquiterpene synthase, HMGR, farnesyl diphosphate synthase 1	Terpenoid biosynthesis	[80]
			pmi-miR6300			
*Ginkgo biloba* L.	Maidenhair tree	Ginkgoaceae	miR_2924, miR_218	HMGS, MK, MPDC, LPS	Terpene trilactones (TTLs) biosynthesis	[81]
			miR_2642			
			miR159a, miR159, miR_2432	LPS	Terpene trilactones (TTLs) biosynthesis	
*Artemisia annua* Pall.	Sweet wormwood	Asteraceae	miR159, miR172	Cytochrome P450 reductase; CYP71AV1 catalyzes	Artemisinin (ART) biosynthesis (sesquiterpene)	[82]
			miR166			
*Ferula gummosa* Boiss.	Ferula	Apiaceae	miR1533, miR5021, miR5658	SPL7, SPL11, ATHB13	Terpene biosynthesis	[76]
*Stevia rebaudiana* (Bertoni) Bertoni	Candyleaf	Asteraceae	miRstv_7^∗^	UDP-glycosyl transferase76G1 (UGT76G1)	Steviol glycoside biosynthesis	[83]
			miRstv_7^∗^	Kaurenoic acid hydroxylase (KAH)	Steviol glycoside biosynthesis	
			miRstv_7^∗^	Kaurene oxidase (KO)	Steviol glycoside biosynthesis	

**Table 3:** Alkaloid and other N-containing metabolites and miRNA.

Botanical name	Common name	Family	miRNA	Target	Function	References
*Nicotiana tabacum* L.	Tobacco	Solanaceae	miRX13^∗^	Putrescine methyltransferase 2 (PMT2)	Nicotine biosynthesis	[50]
			miRX17^∗^	Quinolinate phosphoribosyl transferase 1 (QPT1)		
			miRX20^∗^	Cytochrome P450 monooxygenase (CYP82E4)		
			miRX27^∗^	Quinolinate phosphoribosyl-transferase 2 (QPT2)		
			miR164	NtNAC-R1		[91]
			miRX17, miRX27	QPT1, QPT2, CYP82E4, PMT2		[50]
			miRX20, miRX19			
*Papaver somniferum* L.	Opium poppy	Papaveraceae	miR13	7-O-Methyltransferase (7-OMT)	BIA biosynthesis	[92]
			miR408	Reticuline oxidase		
			miR2161	4′-O-ethyltransferase 2 (4-OMT)		
			pso-miR13	7-O-methyltransferase, S-adenosyl-l-methionine:3′-hydroxy-N-methylcoclaurine 4′-O-methyltransferase 2/4′-O-methyltransferase2 (4-OMT)/FAD-binding and BBE domain-containing protein		
			pso-miR2161			
			pso-miR408			
			pso-miR13	7-OMT	Morphinan	
*Taxus baccata* L.	English yew	Taxaceae	miR164	Taxane 13*α* hydroxylase	Taxol	[93]
*Catharanthus roseus* (L.) G.Don	Madagascar periwinkle	Apocynaceae	miR-5021	Two enzymes involved in biosynthesis of TIAs	Indole alkaloids	[74]
				UDP-glucose iridoid glucosyltransferase		
			cro-miR160, cro-miR164a, cro-miR164b	CrARF16, *ORCA3*, *ORCA4*, and *BIS1*, in TIA biosynthetic pathway	Indole	[95]
			cro-miR393d		Alkaloid biosynthesis	
*Arabidopsis thaliana* (L.) Heynh.	Thale cress	Brassicaceae	miR393	Auxin receptor	Camalexin	[96]
*Curcuma longa* L.	Turmeric	Zingiberaceae	miR5021	Aspartate transaminase, aromatic-amino-acid transaminase	Isoquinoline alkaloid biosynthesis	[48]
*Helianthus annuus* L.	Sunflower	Asteraceae	U3938865 and U4351355	MYB34	Glucosinolates and camalexin (indole alkaloid) biosynthesis	[2]

**Table 4:** Fatty acids, other compound biosynthesis, and miRNA.

Botanical name	Common name	Family	miRNA	Target	Function	References
*Lonicera japonica* Thunb.	Honeysuckle	Caprifoliaceae	U436803	Acyl-CoA synthetase, acyl carrier protein, fatty acid hydroxylase	Fatty acid biosynthesis	[2]
			U977315			
			U805963			
*Paeonia × suffruticosa* Andrews	Tree peony	Paeoniaceae	miR156 b	1-Acyl-sn-glycerol-3-phosphate acyltransferase	Fatty acid biosynthesis	[98]
*Corynespora cassiicola*	Cucumber	Corynesporascaceae	Novel-miR1	4-Coumarate: CoA ligase,	Phenylpropanoid synthesis	[15]
		Novel-miR1-novel-miR7	Novel-miR2	S-Adenosylmethionine decarboxylase		
			Novel-miR3	Peroxidase		
			Novel-miR4	Phosphoenolpyruvate carboxylase		
			Novel-miR5	Pleiotropic drug resistance protein		
			Novel-miR6	Xyloglucan endotransglucosylase/hydrolase family		
			Novel-miR7	Phenylalanine ammonia-lyase		
*Podophyllum hexandrum* Royle	May apple	Berberidaceae	miR2673a	MYB F1, WRKY37		[99]
			miR396b	Flavonol synthase		
				UDP glycosyltransferase		
*Withania somnifera* (L.) Dunal	Ashwagandha	Solanaceae	miR5140	Cycloartenol synthase (CAS1), sterol delta-7 reductase 1	Withanolide biosynthesis	[100]
			miR159	CYP82G		
			miR477	Zeatin o-glycosyl transferase (UGTs)		
			miR530	Secoisolariciresinol dehydrogenase (ABA2)		
*Nicotiana tabacum* L.	Tobacco	Solanaceae	miRn24	Branched-chain amino acid transaminase 3 (BCAT3)	Glucosinolate biosynthesis	[53]
*Arabidopsis thaliana* (L.) Heynh.	Thale cress	Brassicaceae	miR826	Alkenyl hydroxyalkyl producing 2 (AOP2)	Transgenic approach	[49, 101]
			miR5090∗			
*Swertia chirayita* (Roxb.) Buch.-Ham. ex C.B.Clarke	Chiretta	Gentianaceae	miR-168	Acetyl-CoA acetyltransferase (AACT), aspartate aminotransferase (PHAT) premnaspirodiene oxygenase (PSO)	Secondary metabolites biosynthesis	[23]
			miR-11320 miR166a	Ribulose-phosphate 3-epimerase (RPE)		
			miR-11071 miR-156a miR-166b	Phosphoglycerate mutase (PGM)		
				Gene encoding homeobox-leucine zipper protein (HD-ZIP)		
*Arabidopsis thaliana* (L.) Heynh.	Thale cress	Brassicaceae	miR826	Alkenyl hydroxyalkyl producing 2 (AOP2)	Glucosinolate biosynthesis	[49]
			miR5090			
*Solanum tuberosum* L.	Potato	Solanaceae	miR6023, miR6024 and miR6027	Alkaloid metabolism, UMP salvage, lipid biosynthesis, and cellulose catabolism; glycoalkaloid metabolism via JA signaling pathway	UDP-glucose biosynthesis	[34]
*Salvia miltiorrhiza* Bunge	Red sage	Lamiaceae	miR5072	Acetyl-CoA	Tanshinones biosynthesis	[78]
				C-acetyl transferase		
*Stevia rebaudiana* (Bertoni) Bertoni	Candyleaf	Asteraceae	miR319g miRstv_11	KO, UGT85C2, KS, KAH	Steviol glycoside biosynthesis	[83]
*Picrorhiza kurroa* Royle ex Benth.	Kutki	Plantaginaceae	miRNA-4995	One enzyme involved in biosynthesis of terpenoids	Picroside-I (iridoid glycoside)	[73]

## Data Availability

The data supporting this review are from previously reported studies and datasets, which have been cited. The processed data are available from the corresponding author upon request.

## Abbreviations

RISC: Ribonucleoprotein RNA-induced silencing complex
AACT: Acetyl-CoA acetyltransferase
PSO: Premnaspirodiene oxygenase
RPE: Ribulose-phosphate 3-epimerase
PGM: Phosphoglycerate mutase
HD-ZIP: Omeobox-leucine zipper protein
TMV: Tobacco mosaic virus
DCL1: RNAase dicer-like 1
HYL1: Hyponastic leaves
miRNAs: MicroRNAs
SE: Serrate
RNAPII: RNA polymerase II
HEN1: HUA enhancer 1
3′ UTR: 3′ Untranslated region
CDC5: Cell division cycle 5
SPL: Squamosa promoter targeting protein like
MyB: Myeloblastosis
DMAPP: Dimethylallyl diphosphate
IPP: Isopentenyl diphosphate
bAS: Beta-amyrin synthase
CASI: Cycloartenol synthase
QPT2: Quinolinate phosphoribosyl-transferase 2
PMT2: Putrescine methyltransferase 2
BIA: Benzyliso quinoline alkaloids
CyP82E4: Cytochrome P450 monooxygenase
QPT1: Quinolinate Phosphoribosyltransferase 1.
